# Radiofrequency guidewire-facilitated recanalization of chronic thoracic central venous occlusions in hemodialysis patients

**DOI:** 10.1186/s42155-023-00422-6

**Published:** 2024-01-12

**Authors:** Sherif Moawad, Ansar Z. Vance, Ryan M. Cobb, Mark P. Mantell, Raphael Cohen, Timothy W. I. Clark

**Affiliations:** 1 Section of Interventional Radiology, Department of Radiology, University of Pennsylvania Perelman School of Medicine, Philadelphia, PA USA; 2 Division of Vascular Surgery, Department of Surgery, Penn Presbyterian Medical Center, University of Pennsylvania, Philadelphia, PA USA; 3 Division of Nephrology, Department of Medicine, Penn Presbyterian Medical Center, University of Pennsylvania, Philadelphia, PA USA

## Abstract

**Purpose:**

To assess the outcome and safety of radiofrequency (RF) wire recanalization in patients with end-stage renal disease (ESRD) and chronic central venous occlusions (CVO).

**Materials and Methods:**

A retrospective review of ESRD patients who underwent RF-wire recanalization of symptomatic chronic thoracic CVO from January 2017 to August 2022 yielded 20 patients who underwent 21 procedures. All patients had undergone at least one prior unsuccessful attempt at central venous recanalization using conventional catheter-based techniques. Technical success was defined by the ability to cross the CVO using RF-wire recanalization enabling endovascular treatment. Access circuit patency was evaluated based on follow-up imaging and symptomatic improvement.

**Results:**

Radiofrequency wire recanalization was successful in 17/21 procedures (81%) with all patients (100%) reporting resolution of arm ± facial swelling. Three major complications occurred (14%): two hemothoraces and one hemopericardium. Medial stent diameter was 13 mm (range, 9–14 mm). Mean duration of hospital stay was 2 days ± 3 days. Mean procedure time was 158 ± 46 min with a mean fluoroscopy time of 31.7 ± 16.3 min. Primary unassisted patency at 6 and 12 months was 94 ± 6% and 85 ± 10%, respectively. Additional interventions resulted in significantly increased stent graft patency (*P* = 0.006).

**Conclusion:**

Radiofrequency wire-enabled recanalization of CVO in symptomatic dialysis patients has a high rate of technical success with resolution of arm and facial swelling and resumed use of the ipsilateral dialysis access. Although a superior safety profile was seen than with needle-based techniques such as sharp recanalization, major complications were not infrequent indicating that this RF-wire procedure should be performed in centers equipped to manage central venous perforations.

## Introduction

The majority of patients initiating hemodialysis in the United States do so with a tunneled central venous catheter while awaiting placement and/or maturation of a working arteriovenous (AV) access. Although tunneled dialysis catheters remain an important bridge to surgical AV access, the complications of these devices can lead to eventual central venous stenosis and occlusion.

Chronic CVO remains a significant source of morbidity among hemodialysis patients occurring in approximately 25–30%, and can manifest with debilitating arm swelling, inability to utilize the existing AV access, and neck and facial swelling [1–3]. While conventional catheter-based techniques are often successful in crossing these occlusions to enable angioplasty with or without stenting, these techniques fail in a significant subset of these patients, 11–24% [4, 5]. Previously this subset of patients was left with few remaining treatment options, and additional endovascular technologies are needed to increase the spectrum of patients who can continue to use their access without the morbidity and pain of limb and facial swelling.

The RF-wire technology (PowerWire, Baylis Medical, Toronto, Ontario, Canada) has been described as a means of crossing a chronic central thoracic venous occlusion under fluoroscopic and angiographic guidance, followed by conventional angioplasty and stent placement for successful recanalization. This study reports experience with the PowerWire device from a large academic medical center which serves as a regional referral center for hemodialysis patients with complex vascular access.

## Patients and methods

This study was approved by the local institutional review board and was in accordance with the Health Insurance Portability and Accountability Act; informed consent was not required for this type of retrospective study. Between January 2017 and August 2022, a consecutive cohort of 21 procedures in 20 patients was identified from a prospectively maintained quality assurance database; this cohort underwent elective RF-wire facilitated thoracic central venous recanalization. All patients had undergone one or more unsuccessful attempts at central venous recanalization using conventional catheter-based techniques at the authors’ or an outside institution.

A similar clinical protocol was utilized in all patients. Patients were seen in the IR clinic prior to the RF-wire procedure. During that encounter, a detailed review of arteriovenous access history and relevant imaging was performed, and patients and family members counseled on the potential risks of the procedure, including hemothorax, pneumothorax, cardiac tamponade and death. Since all patients had failed prior attempts at central venous recanalization, the venographic images from the prior procedure(s) were sufficient for procedural planning so that CT venography was not obtained.

All procedures were performed with general or monitored anesthesia care, at the discretion of the anesthesiologist. Pre-procedural type and screening was performed in anticipation of potential initiation of a rapid transfusion protocol. Two large-bore sites of IV access were established and a radial artery monitoring line placed with ultrasound guidance.

The RF-wire recanalization technique was performed as follows. Femoral access was established with placement of a 70 cm 7-French sheath in the right atrium (Cook Medical, Bloomington, IN). A directional catheter was advanced through the sheath to the leading edge of the central venous occlusion (superior vena cava, brachiocephalic vein or subclavian vein). The arteriovenous access was punctured and a 6 French angled sheath (Ansel sheath, Cook Medical, Bloomington, IN or Destination sheath, Terumo, Somerset, NJ) placed near the peripheral edge of the central venous occlusion. Through the peripheral sheath and the central venous directional catheter, simultaneous injections of contrast were performed with digital subtraction angiography (DSA) to delineate the length and anatomy of the central venous occlusion. At the discretion of the operator, an ‘outside-in’ or ‘inside-out’ strategy was then selected depending on the geometry of the occlusion. In general, the side of the occlusion with a venographically tapered edge or ‘beak’ was selected as the site of RF-wire recanalization.

An Amplatz-type snare (One-Snare, Merit Medical, South Jordan, UT) or tri-petal snare (En-Snare, Merit) was positioned on the opposite side of the occlusion. A 4-French or 5-French angled-tip catheter was advanced over a wire to the leading edge of the occlusion. Through this, an 0.035-in, 110-g tip Baylis RF guidewire (Powerwire) was advanced through the angled-tip catheter to the edge of the occlusion. Straight-tip or 40-degree angled tip PowerWire selection was at the discretion of the operator.

Using 1 or 2-s pulses of RF energy, the RF-wire was advanced in 2-mm increments across the occlusion with fluoroscopic guidance. Triangulation techniques were utilized after each second or third pass of the RF-wire to ensure intended traversal of the occlusion was occurring; these consisted of 30 to 50 degree oblique and contralateral oblique static and dynamic fluoroscopic views after each 5 mm excursion of the RF-wire. Although all procedures were performed in a biplane angiography suite equipped with cone-beam CT (Artis, Siemens, Erlangen, Germany) it was seldom possible to perform cone-beam CT due to positioning of the patient and ventilatory equipment.

Contrast injections through the directional catheter were used when position of the RF-wire in relation to the snare on the opposite side of the occlusion could not be ascertained with orthogonal, steep oblique fluoroscopic views. When necessary, the RF wire was withdrawn and advanced across the occlusion using a different tract. Successful crossing of the occlusion was confirmed by visualization of the RF wire within or beyond the snare, at which point the RF wire was retrieved with the snare and withdrawn through the sheath to establish though-and-through guidewire access spanning from the site of femoral access to the site of upper extremity and/or internal jugular access. A 4 French, 100 cm long Berenstein catheter was then advanced over the RF-wire through both sheaths, and the RF-wire exchanged for a 260 cm 0.035-in Amplatz wire.

**Fig. 1:**
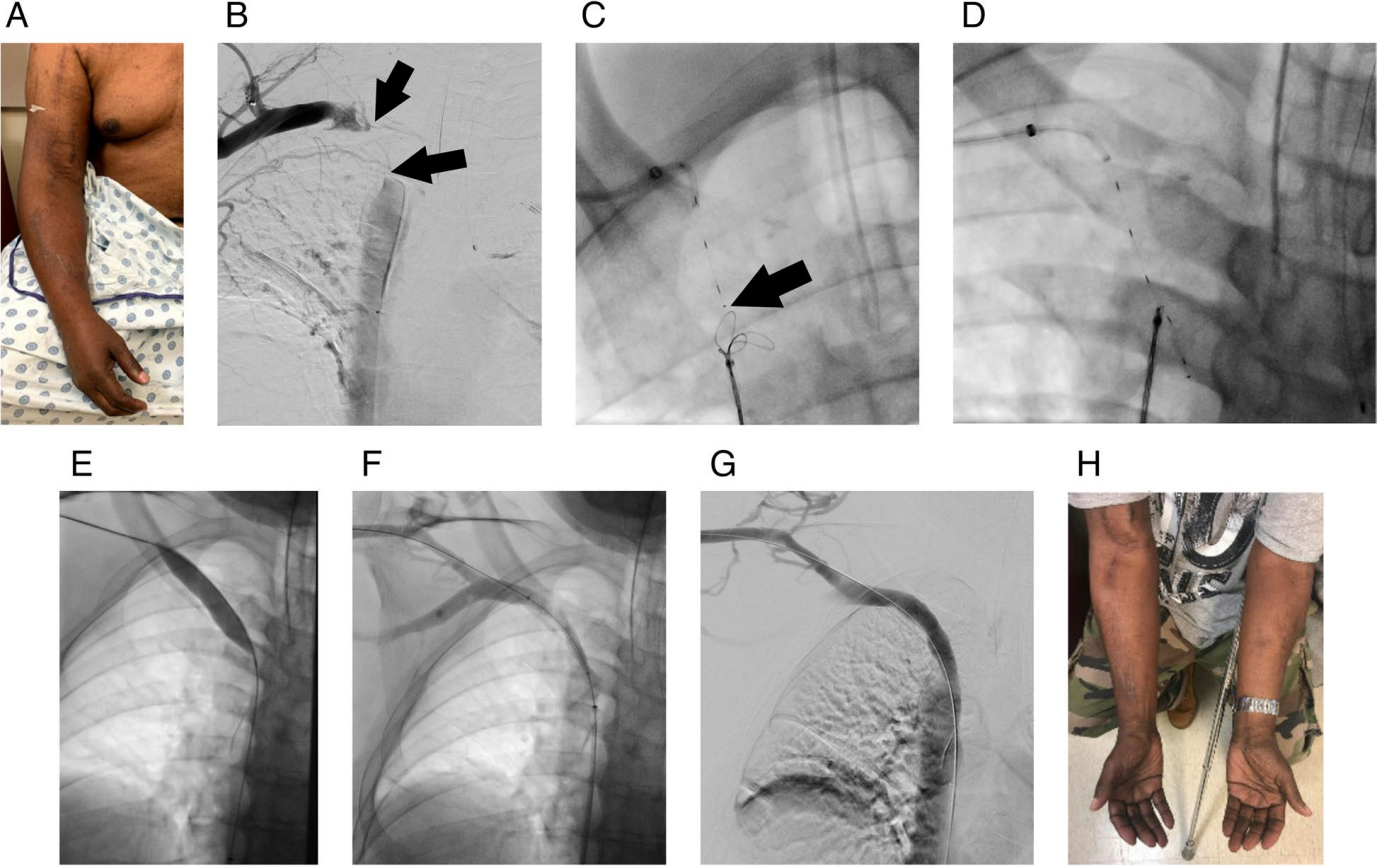
66-year old male on hemodialysis with multiple failed prior access sites and massive arm swelling. He had a poorly functioning right transposed brachiocephalic fistula with high venous pressures and prolonged bleeding after needle decannulation secondary to chronic brachiocephalic occlusion. **A** Initial IR clinic visit showing right arm swelling. **B** Initial venograms performed through the right brachiocephalic fistula and the superior vena cava showing chronic occlusion of the brachiocephalic vein (arrow). **C** Left anterior oblique vein during triangulation of PowerWire (tip shown with arrow) across the occlusion toward the snare in the superior vena cava. **D** Capture of the PowerWire with the snare. **E** Balloon dilation of the brachiocephalic occlusion to 14 mm (performed following serial dilation from 6/10/12 diameter balloons, with interval venograms). **F** Venography during positioning of a 13 mm × 50 mm PTFE-lined stent graft (Viabahn, Flagstaff, AZ) across the site of elastic recoil following 14 mm venoplasty. **G** Completion venogram following stent deployment and post dilation with a 14 mm balloon. **H** IR clinic visit at 4 weeks following interval resolution of both arm swelling and access site dysfunction

**Fig. 2:**
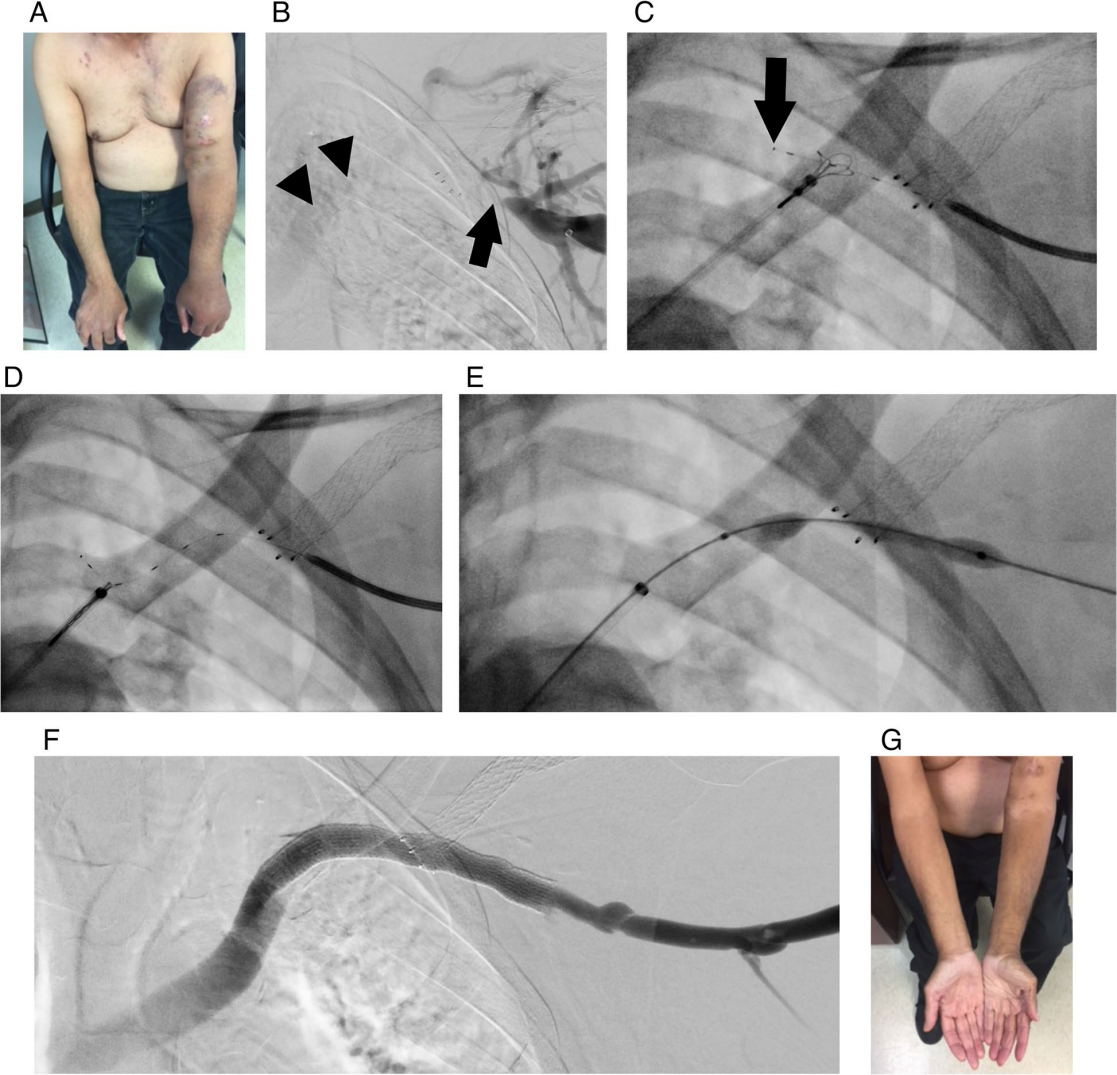
67-year old male on hemodialysis with multiple failed prior access sites and a poorly functioning left transposed brachiobasilic fistula with high venous pressures. He has a chronic left axillosubclavian vein occlusion, secondary to a bare nitinol stent placed within the cephalic arch at an outside facility at the time of a previously functioning brachiocephalic fistula which has since been abandoned. **A** Initial IR clinic visit showing left arm swelling and hyperpigmentation from chronic venous hypertension. **B** Initial venogram performed through the left brachiobasilic fistula showing chronic occlusion of the axillary vein at the site of the prior cephalic arch stent (arrow) with mediastinal collaterals reconstituting the left brachiocephalic vein (arrowheads). **C** Fluoroscopic view after traversal of PowerWire (tip shown with arrow) across the occlusion into the snare in the left subclavian vein. **D** Capture of the PowerWire with the snare. **E** Initial balloon dilation of the axillosubclavian occlusion with an 8 mm ultrahigh pressure balloon (Conquest, Becton Dickinson, Franklin Lakes, NJ). **F** Completion venogram following serial balloon dilation to 10 mm, deployment of a 10 mm × 100 mm PTFE-lined stent graft (Viabahn) and 10 mm venoplasty. **G** IR clinic visit at 4 weeks showing complete resolution of arm swelling

Sequential dilation of the occlusion was performed in 2-mm increments beginning with a 6-mm diameter balloon. Venograms were performed after each balloon angioplasty until a diameter of 12 mm had been obtained. After upsizing the upper extremity sheath to 11-French (Terumo), an polytetrafluoroethylene (PTFE)-covered stent graft (typically 13 mm in diameter, Viabahn, Gore, Flagstaff, Arizona) was deployed across the occlusion and post-dilated with a 12-mm or 14-mm balloon; a single patient underwent placement of a 8 mm × 29 mm Gore VBX stent graft post-dilated to 14 mm. Completion venograms were performed and if venography showed that a portion of the recanalized central venous segment remained unstented, an additional overlapping stent graft was placed, balloon dilated and additional completion venograms performed. Figures 1 and 2 show representative examples of right and left sided central venous occlusions from the present series.

Access sheaths were removed and hemostasis achieved with manual compression. Patients remained in hospital overnight. Dual antiplatelet therapy with clopidogrel and acetylsalicylic acid (ASA) was started immediately after the procedure. A chest radiograph and follow-up hemoglobin level were obtained within 6 h of the completion of the procedure, or sooner if the patient developed a change in vital signs or symptoms. Patients were dialyzed the following morning and typically discharged from hospital later that day, and then re-evaluated in the IR clinic within 30 days to confirm resolution of facial, neck and/or arm swelling. Thereafter, patients were seen at 3-, 6- and 12-months following stent graft placement with duplex ultrasound evaluation of the stent construct. Thereafter, patients were seen every 6 months. Patients would continue to receive periodic endovascular interventions on their remaining hemodialysis access circuit at outside facilities as clinically indicated, so it was not possible to determine duration of patency of the remaining dialysis access circuit.

Technical success was defined as successful RF-wire advancement across the thoracic venous occlusion enabling snaring of a through-and-through guidewire to enable endovascular recanalization. Complications were defined in accordance with the Society of Interventional Radiology consensus guidelines [6]. Kaplan Meier estimates of central venous stent construct patency were performed including primary unassisted, primary assisted and secondary patency. Intergroup comparisons of central venous stent patency were performed with the log-rank test.

## Results

Demographics and lesion characteristics of the patient cohort are shown in Table 1. A total of 21 procedures were performed on 20 patients. Technical success was achieved in 17 lesions (81%). There was 1 acute stent thrombosis that required thrombectomy and re-stenting on post procedure day 2. At the 30-day interventional radiology clinic visit, all patients had resolution of arm ± facial swelling. The location of the occlusions and stent placement are shown in Table 2. Two bifurcated stent constructs were required, one which involved right subclavian and bilateral brachiocephalic veins, and the other which recanalized and stented the base of the right internal jugular vein and the right subclavian and brachiocephalic veins. All cases were crossed with an “outside-in” approach except for one. The straight tip RF-wire was used in 12 cases (57%) and the 40° angle RF-wire was used in 8 cases (38%). One lesion required the use of both the straight and angled tip PowerWires. Fourteen cases (67%) were performed under general anesthesia and 7 cases (33%) performed under monitored anesthesia care (MAC). Mean duration of hospital stay was 2 days ± 3 days. Mean procedure time was 158 ± 46 min with a mean fluoroscopy time of 31.7 ± 16.3 min. Mean contrast volume used during the procedure was 70 mL.

**Table 1:** Patient demographics and lesion characteristics, N, (%)

Characteristic	Value
**Sex**	
Male	11 (55)
Female	9 (45)
**Age**	
Mean	64.0 (SD 8.6)
Range	50–78
**AV Access type**	
Fistula	14 (74%)
Graft	5 (26%)
**Laterality**	
Right	14 (70%)
Left	5 (25%)
Central	1 (5%)
**Main indication for intervention**	
Arm Swelling	18 (90%)
Face/neck swelling	2 (10%)

**Table 2:** Stent placement location in 17 successful recanalization procedures

Stent location	N
BCV only	6
Border of BCV and SCV	7
Border of SCV and AV	1
Bifurcated stent construct – IJ and BCV	1
Bifurcated stent construct – R and L BCV	1
SVC	1

*BCV* Brachiocephalic vein, *SCV* Subclavian vein, *AV* Axillary vein, *SVC* Superior vena cava

**Fig. 3:**
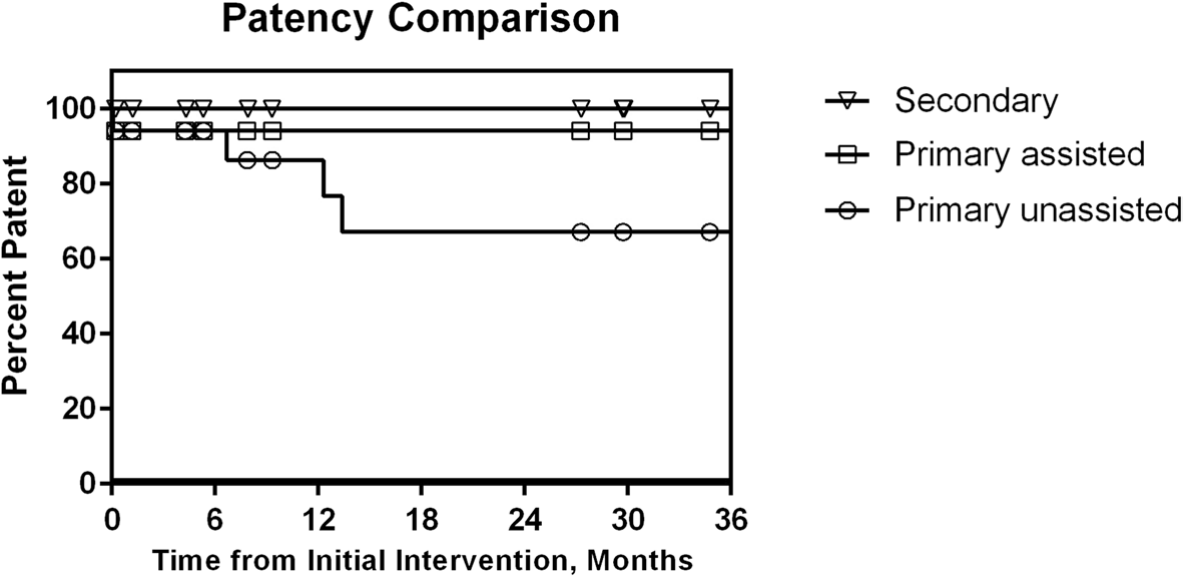
Kaplan Meier curve showing access circuit primary unassisted, primary assisted and secondary patency in the study cohort following RF-wire enabled recanalization

The mean length of occlusion was 3.6 cm ± 1.6 and with occlusion length ranging from 0.5 cm to 7.2 cm. The median PTFE-covered stent graft diameter was 13 mm (range 9–14 mm). The mean follow-up was 827 days with primary unassisted patency of 94 ± 6% and 85 ± 10% at 6 and 12 months, respectively. Additional interventions including angioplasty (8 procedures in 4 patients), restenting (*n* = 2) and thrombectomy (*n* = 1) resulted in significantly increased primary assisted and secondary stent graft patency (*P* = 0.006); secondary patency was 100% at 36 months (Fig. 3).

**Fig. 4:**
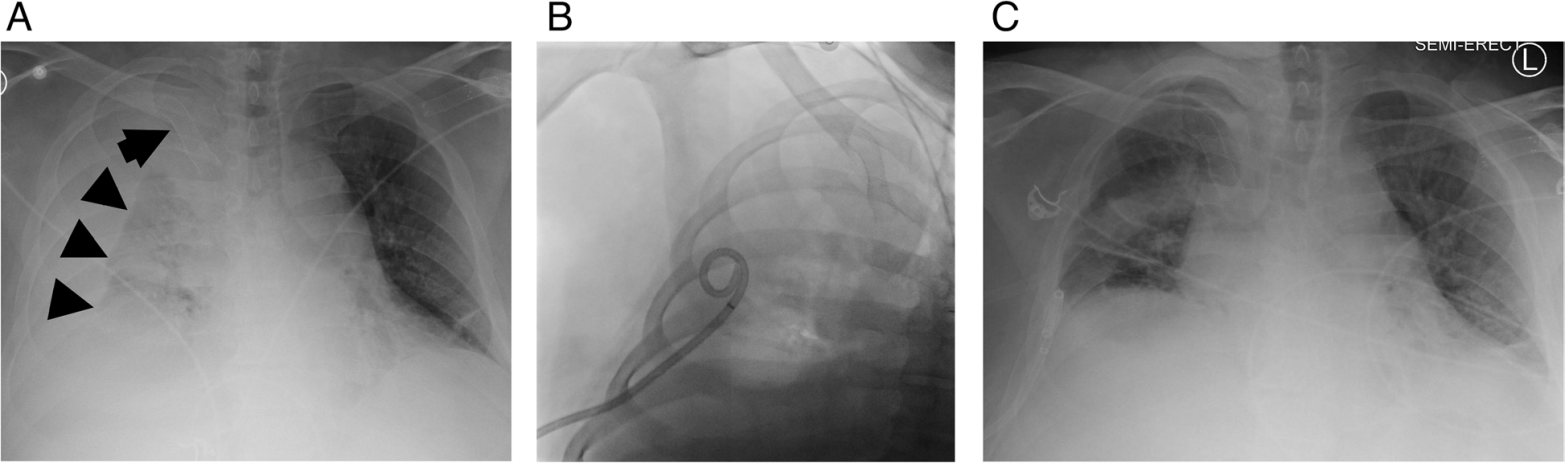
Complication following successful RF-wire enabled recanalization and stenting of a right brachiocephalic occlusion. **A** Portable chest radiograph obtained immediately following procedure showing stent graft in good position (arrow) but the presence of a large right hemothorax (arrowheads). **B** Locking loop 14 French chest tube was placed in the IR suite, yielding 800 mL of blood. **C** Chest radiograph obtained 48 h later showing resolution of hemothorax. The chest tube was subsequently capped and then removed

There were 3 major complications (14%) in the current study. Two patients developed a hemothorax ipsilateral to the side of the CVO managed with image-guided chest tube placement and blood transfusion (Fig. 4). One patient had hemopericardium that was managed by percutaneous pericardial drain placement on post operative day 1, and the patient was discharged the following day. One of the hemothorax patients had had a failed attempt initially and deferred returning for a repeat attempt until 15 months later, with successful crossing of the occlusion using the PowerWire device during the second procedure.

## Discussion

Endovascular techniques for recanalization of chronic central venous occlusions among hemodialysis patients continue to evolve. Sharp recanalization utilizes the stiff end of a guidewire or a small-caliber (e.g. Chiba) needle to cross a occlusion [7–9] enabling subsequent angioplasty and stenting. Extension of this needle-based technique to an integrated crossing needle within the tip of a delivery catheter was reported by Anil and Tenaja using the Outback device [10]. Subsequent technology employed by the Surfacer device (Bluegrass Vascular Technologies, San Antonio, Texas) utilizes a longer needle and a cutaneous radio-opaque targeting marker for crossing right-sided thoracic venous occlusions, enabling tunneled hemodialysis catheter insertion [11, 12]. However, the Surfacer device cannot be used for left-side central venous occlusions nor as a means of venous recanalization and stenting as it does not establish continuity of the subclavian and brachiocephalic veins. Radiofrequency wire recanalization may provide a more versatile technology to treat both left- and right-sided occlusions, as well as occlusions that are longer than the excursion length of needle-based devices such as the Surfacer or Outback devices. In the present series, radiofrequency wire recanalization of refractory thoracic central venous occlusions was a relatively safe and effective option. Technical success rate was 81% compared to several prior reports ranging from 50–100%. The current study had a mean occlusion length of 3.6 cm. Kundu et al. reported successful PowerWire crossing of 50% of central venous occlusions with a mean length of 7.3 cm [13]. Sivananthan et al. reported a 69% success in RF-wire crossing of central occlusions in 12 patients; successfully crossed occlusions were shorter (mean length 3 cm) compared to uncrossable occlusions (mean length 9 cm) [14]. In a larger cohort of 42 patients with occlusion lengths ranging from 1.5 cm to 10 cm, Guimaraes et al. reported 100% success in PowerWire crossing with most occlusions (67%) involving the brachiocephalic vein [15]. More recently, Keller et al. reported 80% success in PowerWire crossing of thoracic and infradiaphragmatic central venous occlusions including a cohort of 11 patients with brachiocephalic/SVC occlusions; mean lesion length was 4.9 cm [16].

A major concern of this technology is that the RF wire can pass through virtually any surrounding soft tissue. Another limitation is that the electrical current within the monopolar system is deactivated when the tip of the PowerWire contacts a metal structure such as a prior stent; while this can be advantageous when crossing a native venous occlusion (as the deactivation produces an audible signal, thereby confirming that the RF-wire has established contact with the snare positioned on the distal end of the occlusion), this can make crossing occluded venous stent constructs more challenging. All of the patients in the present series had native venous occlusions with the exception of one patient who had a cephalic arch stent ‘jailing’ the axillosubclavian junction (Fig. 2); in this patient numerous oblique views were necessary to ensure the RF wire did not pass through the interstices of the prior stent.

We experienced 3 major complications during RF-wire enabled recanalization which included two hemothoraces and one hemopericardium. Hemopericardium has been recognized as a known complication on prior studies in which the RF wire inadvertently enters the pericardial space [8, 9]. This event was identified during the procedure using post-traversal venography and treated with pericardial drain placement and close monitoring. Lesion location was at the border of the right brachiocephalic and subclavian veins with a 4.2 cm length of occlusion. The two patients with hemothoraces were managed by chest tube placement and prolonged admission. One of the patients had a hemothorax diagnosed post procedure during recovery after successful crossing and stent placement. Notably, this is the same patient who had an acute in-stent occlusive thrombus (post procedure day 2) that was treated with thrombectomy and extension of the stent construct more peripherally into the axillary vein. This in-stent thrombus may have been attributable to a low flow state given the large hemothorax requiring blood transfusion and intensive care unit admission. In the second patient, hemothorax occurred after a failed RF-wire crossing attempt. A chest CT performed for shortness of breath revealed a moderate-size hemothorax requiring chest tube placement; this patient returned for successful PowerWire crossing after 15 months. Both patients had right sided lesions with lengths of occlusion of 3.6 and 4.2 cm, respectively. Sivananthan et al. [14] had a 69% success rate with 1 fatal complication secondary to tracheal perforation. We did not observe any tracheal perforation in the present series but when long, left-sided brachiocephalic occlusions are being crossed (thereby creating potential for tracheal puncture) frequent triangulation and interval venography are mandatory.

In addition to the initial failure of the patient with the right brachiocephalic occlusion in whom a hemothorax developed after the first procedure, the three additional failures occurred from inability to transverse the central occlusion to establish true lumen re-entry and snaring of the RF-wire. Following each of these three remaining failures, patients were offered a repeat attempt at RF-wire recanalization however each of the patients declined.

All patients with successful recanalization had resolution of arm/facial swelling with preservation of dialysis access in the involved extremity. This is in line with the previously reported literature [13–16].

The primary unassisted patency in the current study was 94 ± 6% and 85 ± 10% at 6 and 12 months respectively. Similarly, Guimaraes et al. demonstrated a 95% patency at 6 and 9 months. However, the reported patency did not specify whether it was primary unassisted, primary assisted or secondary patency [15]. Keller et al. reported primary unassisted patency of 56% at median follow up of 14.1 months [16]. However, restenosis/occlusion was noted in that series to occur less often in patients with ESRD and central venous catheter usage.

This study has a number of limitations, including its retrospective design, small cohort size, absence of long-term follow up and single center experience.

## Conclusions

RF-wire facilitated recanalization demonstrated a high rate of technical success and resolution of arm and facial swelling. This technology enabled resumed use of the ipsilateral dialysis access which is essential for ESRD patients with limited venous reserve. Although a superior safety profile was seen than with needle-based techniques such as sharp recanalization, major complications were not infrequent indicating that this RF-wire procedure should be performed in centers equipped to manage central venous perforations.

## Data Availability

The datasets used and/or analysed during the current study are available from the corresponding author on reasonable request.

## Abbreviations

CVO: Central venous occlusion
AV: Arteriovenous
RF: Radiofrequency
ESRD: End stage renal disease
PTFE: Polytetrafluoroethylene
CT: Computed tomography
ASA: Acetylsalicylic acid
